# Working Women in Large Towns

**Published:** 1890-11-08

**Authors:** 


### Working Women in Large Towns.

### XV.—SOCIAL, MORAL, AND PHYSICAL ASPECTS OF THE SUBJECT.

We have already discussed the question whether it would be well, supposing it were possible, to forbid women to work. But as the subject was then considered merely from an economical point of view, we must return to it for a few moments, and briefly inquire whether such an enactment might be expected to benefit the community socially and morally.

At first sight we might be disposed to give an unqualified Yes to this question. We all feel that a woman's natural place is home; that the ideal woman should have nothing to do with outside labour, but should find her time and thoughts fully occupied in making the home a little haven of rest for the weary breadwinner, in training and caring for the children who cluster round her. And as far as married women are concerned, there can be no doubt that a world in which they were engaged in real "home work" would be a vast improvement on the present state of things. But we must remember that it by no means follows that if a woman has no outside work, she will, as a matter of course, devote herself to the home. Unless she has a taste for domestic duties, she will too often seek to fill up her time and thoughts in ways more harmful than that of which we would deprive her. We hear that in Sheffield, where there is comparatively little female labour, women have taken to gambling; and even if they stop short of this, a gadding, gossiping, scandal-loving wife is no better—nay, is worse—than one who spends her time in honest labour. What our women really want, then, is better training in domestic duties, and a deeper sense of their responsibilities as wives and mothers; and if this can be given them, they will gradually give up other work so far as they can, seeing that what is gained with one hand is too often lost with the other, through the spoiling of the home-life, and the abandonment of the many little domestic economies which save, although they do not make, money.

Then there are the unmarried women and girls to be considered. Theorists—especially theorists about women—are apt to talk as if every woman was a wife and a mother, and as if families remained for ever at one particular stage of their development. The family consists for them of the father, the bread-winner—the mother, the nurse and housekeeper—and some half-dozen curly-headed boys and girls, in pinafores and socks. With this typical family for ever in their minds, they preach and theorise, and would even like to legislate, quite forgetting that the big girls into which some of that family must inevitably grow, deserve special and careful consideration. Whether these girls eventually marry or not, they must work for some years of their life, if, indeed, they are to be kept out of harm's way; for the tiny home which afforded plenty of work and interest for the mother, when her little ones were all about her, cannot supply sufficient employment for her and her growing-up daughters together; and as they themselves have learnt at school, "Satan finds some mischief still, for idle hands to do." Even if men's wages could be so adjusted as to make up, economically speaking, for the loss of their daughters' earnings, the idleness of our girls would be a serious evil. The American Commissioner of Labour mentions a fact which is well worth pondering—that about thirty-two per cent. of the fallen women whose previous circumstances he had inquired into, had come to that life straight from their own homes. Surely it was idleness, rather than work, which was chargeable with the ruin of these poor girls. To the daughters of our artisans and labourers, no less than to the daughters of the well-to-do, is Mrs. Browning's injunction applicable:—

"Get leave to work
In this world—'t'is the best you get at all;
For God in cursing gives us better gifts
Than men in benediction."

But while work is good, certain conditions of that work may be bad; and it certainly behoves everyone who cares for the welfare of our working women to inquire how far such conditions are separable from the work, and what can be done to improve them. It cannot be good, for instance, that wages should be so low as to give certain classes of workers no choice between extreme poverty and a life of sin, whether we consider the matter economically merely, or from social, moral, and, it may be added, physical points of view; and we are therefore furthering the all-round interests of the workers if we help them to secure better wages, by means of combination among themselves. Moreover, it is certain that unions among workers not only protect them from oppression by the employers, but prevent foolish and ill-judged action on the part of the workers themselves, besides promoting among them self-respect and friendliness towards each other.

*(To be continued.)*

